# Comparative metabolomic profiling of women undergoing in vitro fertilization procedures reveals potential infertility-related biomarkers in follicular fluid

**DOI:** 10.1038/s41598-022-24775-5

**Published:** 2022-11-29

**Authors:** Mohamed Ziad Dabaja, Aline Amaro dos Santos, Denise Maria Christofolini, Caio Parente Barbosa, Diogo Noin de Oliveira, Arthur Noin de Oliveira, Carlos Fernando Odir Rodrigues Melo, Tatiane Melina Guerreiro, Rodrigo Ramos Catharino

**Affiliations:** 1 Innovare Biomarkers Laboratory, School of Pharmaceutical Sciences, University of Campinas, Rua Cinco de Junho, 350, Cidade Universitária Zeferino Vaz, Campinas, SP 13083-970 Brazil; 2 Instituto Ideia Fértil de Saúde Reprodutiva, Santo André, SP 09060-650 Brazil; 3 Centro Universitário FMABC, Santo André, SP 09060-870 Brazil

**Keywords:** Biochemistry, Biomarkers, Diseases

## Abstract

Infertility is a worldwide concern, affecting one in six couples throughout their reproductive period. Therefore, enhancing the clinical tools available to identify the causes of infertility may save time, money, and emotional distress for the involved parties. This study aims to annotate potential biomarkers in follicular fluid that are negatively affecting pregnancy outcomes in women suffering infertility-related diseases such as endometriosis, tuboperitoneal factor, uterine factor, and unexplained infertility, using a metabolomics approach through high-resolution mass spectrometry. Follicular fluid samples collected from women who have the abovementioned diseases and managed to become pregnant after in vitro fertilization procedures [control group (CT)] were metabolically compared with those from women who suffer from the same diseases and could not get pregnant after the same treatment [infertile group (IF)]. Mass spectrometry analysis indicated 10 statistically relevant differential metabolites in the IF group, including phosphatidic acids, phosphatidylethanolamines, phosphatidylcholines, phosphatidylinositol, glucosylceramides, and 1-hydroxyvitamin D3 3-d-glucopyranoside. These metabolites are associated with cell signaling, cell proliferation, inflammation, oncogenesis, and apoptosis, and linked to infertility problems. Our results indicate that understanding the IF’s metabolic profile may result in a faster and more assertive female infertility diagnosis, lowering the costs, and increasing the probability of a positive pregnancy outcome.

## Introduction

Infertility is the inability to achieve a successful pregnancy after 12 months or more of unprotected sexual intercourse without contraceptive methods^1^. According to the National Institute of Health and Human Development (NIH), 15% of couples at reproductive age may have infertility-related problems, although the exact prevalence is difficult to estimate. Infertility is not a gender-linked issue; however, women account for 60% of the cases^2^. Furthermore, although infertility is a composite factor (man and woman combined), a cultural construction has been that the act of conceiving children is mainly attributed to women, which explains why the majority of fertility assessments are aimed at them^3^. Assisted reproduction techniques include artificial insemination (AI) and intracytoplasmic sperm injection (ICSI) and have been responsible for the birth of more than five million children. In 2008, 61,430 newborns were conceived using these techniques, and in 2014, 190,394 treatment cycles were started, indicating the enormous potential and demand for technologies that help couples with infertility problems^4^. The technique of AI is more prevalently used when compared with the use of ICSI. AI technique research and clinical uses have advanced over the last decades. The percentage of success per treatment cycle is only 40%, accompanied by a cost of approximately 10,000 US dollars, resulting in exacerbated expenses without a guaranteed successful outcome. Furthermore, technique success is affected by many factors, such as age, height, weight, genetic abnormalities, environmental factors, hormonal dysregulation, reproductive history, and lifestyle. However, it is directly linked to clinically diagnosed diseases^5^.

Endometriosis is a gynecological disease that affects 15% of women of childbearing potential, causing pelvic/abdominal pain and infertility-related complications. In addition, 20% of cases can be completely asymptomatic. Endometriosis is characterized by the extension of endometrial growth outside the uterine cavity. It manifests through mechanisms that include genetic, immunological, and environmental factors^6^. Another leading cause of female infertility, tuboperitoneal factor, is an anatomical anomaly of the fallopian tubes that affects between 25 and 35% of women with infertility problems^7^. The most frequent causes of fallopian tube anatomy alterations include tubal surgery, acute perforated appendicitis, septic abortion, and pelvic inflammatory disease^8^. Uterine factors, such as fibroids, polyps, and adhesions, complete the list of significant causes of infertility in clinically diagnosed women, affecting 20–50% of women of childbearing potential. Submucosal and intramural fibroids affect the fertility rate in women with natural and in vitro fertilization (IVF)^9^. In addition, the egg implantation process is directly affected when fibroids are situated in the intrauterine cavity, resulting in the impairment of the pregnancy process. Although current technologies are well-established and advanced, the identification and clinical diagnosis of these diseases are not assertive, creating doubts and misdiagnoses^10^. In addition, there are cases of unexplained infertility, i.e., a lack of clinical diagnosis in women who have gone through all the standard investigations tried to become pregnant for more than 12 months through unprotected sexual intercourse or assisted reproduction techniques, and were not successful^11^.

Human follicular fluid provides the environment for follicle development and oocyte maturation and contributes to oocyte quality and IVF outcome. It is composed of proteins, steroid hormones, lipids, and other metabolites, as observed in “Omics” based studies^12^. The components of follicular fluid may have several origins: secretions from granulosa cells, theca cells, oocytes, and blood plasma composition transferred through the thecal capillaries^13^. Despite its enormous potential to predict oocyte quality and composition, follicular fluid is still underused, and its metabolic profile remains largely unexplored.

Metabolomics, or metabolomic profiling, is defined as the quantitative measurement of a large number of low molecular weight molecules within a particular sample, including bodily fluids (urine, blood, saliva), tissues, and breath exhalate^14^. Metabolomic profiling is a powerful tool to predict and measure the biochemical activities within cells, and it has proven to be useful in disease screening, diagnosis, characterization, and monitoring^15^. A metabolomic biomarker is a biological observation that could be a single molecule, as well as a pattern of several molecules that can anticipate a clinically relevant endpoint^16^. Biomarkers are usually measured over a shorter period and are easier and less expensive than direct measurements.

Endometriosis, tuboperitoneal factor, uterine factor, and unexplained infertility represent up to 70% of clinical diagnoses and are the leading causes of female infertility. However, some women diagnosed with these abovementioned diseases can still achieve pregnancy after IVF procedures while others can not. Therefore, understanding the differences between the metabolic profiles of women that could not achieve pregnancy and the metabolic profiles of women that were able to become pregnant, after IVF procedures, becomes of paramount importance for increasing the chances of positive pregnancy outcomes. Thus, considering the gap in metabolic information regarding biomarkers that are negatively affecting fertility in IVF procedures, we performed a metabolomics study to observe if any infertility-related biomarkers could be detected in the follicular fluid. The results demonstrate the possibility of using 10 statistically relevant molecules to develop and improve women`s infertility diagnosis, enhancing clinical and treatment assertiveness and, consequently enhancing IVF outcomes.

## Methods

### Study design

Follicular fluid was obtained from ovarian punctures of the three largest follicles of women with infertility complaints, undergoing highly complex assisted reproduction procedures (oocyte retrieval and ICSI). Exclusion criteria were age ≥ 37 years old, body mass index (BMI) < 18.5 and > 30, significant blood level in the follicular fluid samples making its analysis unfeasible, autoimmune diseases, karyotype alterations or diagnosed monogenic diseases, and ovulatory dysfunctions.

The cause of infertility was investigated according to the propaedeutic for infertile couples: serological tests, hormonal and biochemical profiles, testing for sexually transmitted diseases, imaging tests, genetic and immunological abnormalities, seminal analysis, hysterosalpingography, hysteroscopy, and laparoscopy. Based on this investigation, the medical team of Ideia Fertil attributed the cause of the couple’s infertility. Patients whose infertility factors were classified as endometriosis, tuboperitoneal factor, uterine factor, and unexplained infertility were included in the research project. Inclusion criteria in each research group are:

- Unexplained or idiopathic infertility: absence of changes in tests performed in infertility investigation.
- Tuboperitoneal factor: tubal ligation and/or presence of a tubal obstruction in one or both tubes.
- Uterine factor: the presence of fibroids, polyps, and/or endometrial synechia.
- Endometriosis: lesions on laparoscopy or laparotomy, confirmed by pathological analysis. The staging of the condition has been established by the guidelines of the American Society for Reproductive Medicine (ASRM, 1997) and histological confirmation of the disease.

Pregnancy results were used to classify the participants as the infertile (IF) or control (CT) group. Pregnancy results were confirmed by serum measurement of the beta fraction of hCG (βhCG) from the 12th day after embryo transfer.

In total, 49 women were recruited, with 25 classified as the IF group and subclassified in the unexplained infertility (9), tuboperitoneal factor (8), uterine factor (5), and endometriosis (3) groups, and 24 classified as the control (CT) group and subclassified in unexplained infertility (7), tuboperitoneal factor (8), uterine factor (5) and endometriosis (4) groups.

### Controlled ovarian hyperstimulation

The controlled ovarian hyperstimulation protocol was performed as described by Barbosa et al.^17^.

### Mass spectrometry analysis

Samples were prepared by diluting 10 µL of follicular fluid in 990 µL of methanol. Then, the samples were centrifuged at 3500 × rpm for 5 min at 4 °C. The aliquot of 1000 µL of the supernatant was ionized with 0.5 µL of formic acid prior to direct infusion in a high-resolution mass spectrometer (ESI-LTQ-XL Orbitrap Discovery, Thermo Fisher Scientific, Bremen, Germany) with a resolution of 30,000 FWHM. Each sample was analyzed five times with 30 s for each acquisition. Data were acquired in the positive ion mode and in the mass range from 500 to 2000 m*/z*. The parameters used were: sheath gas at 5 (arbitrary units), flow rate of 10 μL min^−1^, spray voltage of 5 kV, and capillary temperature of 250 °C.

### Statistical data analysis and metabolite annotation

Mass spectra data of the patients who became pregnant (CT) were compared with that of the patients who did not become pregnant (IF) within individual datasets of each cause of infertility: endometriosis, tuboperitoneal factor, uterine factor, and unexplained infertility. The data obtained were treated by multivariate statistical analysis using MetaboAnalyst 4.0 online software (www.metaboanalyst.ca); Ortho-PLS-DA was used to choose the characteristic markers of each group involved in the analysis. Only candidate markers with area under the curve (AUC) scores equal to, or greater than, 0.9 were evaluated. The structural elucidations of the markers were obtained by comparing the experimental obtained *m/z* values to its theoretical equivalent mass compatible in the Lipid MAPS online database (University of California, San Diego, CA—www.lipidmaps.org), HMDB version 3.6 (HumanMetabolome database—www.hmdb.ca), and METLIN (Scripps Center for Metabolomics, La Jolla, CA—www.metlin.scripps.edu).

### Ethical approval

Recruitment of patients was conducted at the Instituto Ideia Fértil de Saúde Reprodutiva, Santo André, São Paulo, Brazil, an Institute for reproductive health treatment. The study was conducted according to the guidelines of the Declaration of Helsinki and was approved by the Research Ethics Committee of Centro Universitário FMABC (Committee number 550475).

### Informed consent

Informed consent was obtained from all subjects involved in the study.

## Results

This study aimed to understand the reasons why the IF group could not achieve pregnancy even though the women were diagnosed with the same infertility-related diseases as the CT group. Therefore, to elucidate biochemical mechanisms and causes of infertility, we performed a comparative metabolomics assay between the IF and CT groups through high-resolution electrospray ionization mass spectrometry (ESI-HRMS) analysis.

To determine the metabolites responsible for infertility, metabolomics analysis was performed for biomarker elucidation in the infertile group over the control group, allowing the filtering and analysis of only the metabolites related to infertility and exploration of their biological and biochemical functions.

### Biomarker identification through ESI-HRMS analysis

**Figure 1:**
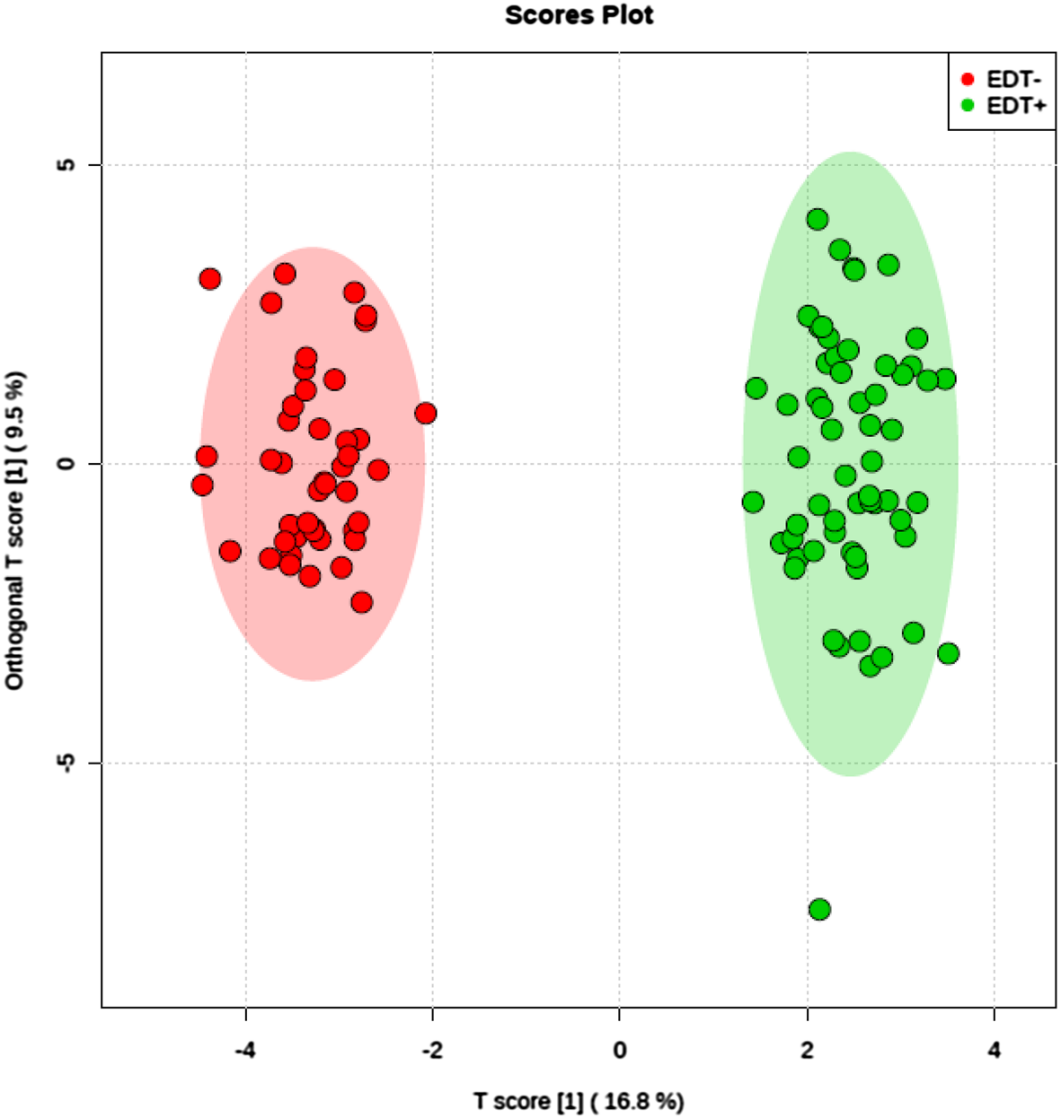
Endometriosis Score Plot. Orthogonal partial least squares discriminant analysis (OPLS-DA) score plot model indicating separation between non-pregnant women (red) and pregnant women (green), both groups diagnosed with endometriosis displayed clustering of data on positive ion mode. Figure generated using MetaboAnalyst (www.metaboanalyst.com). The mass spectrometry analysis was performed in quintuplicate for each sample, therefore each patient is represented by 5 (five) dots.

**Figure 2:**
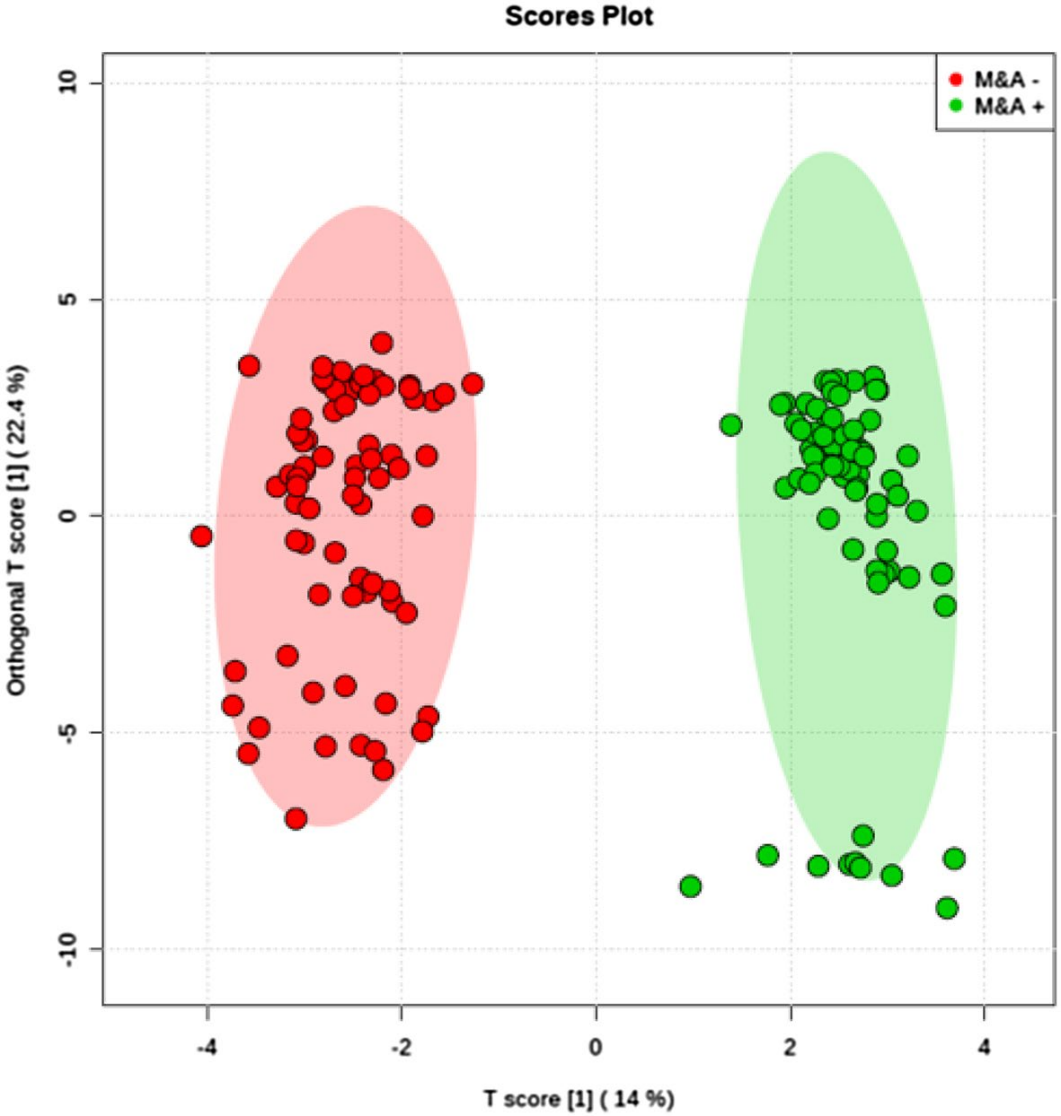
Uterine Factor Score Plot. Orthogonal partial least squares discriminant analysis (OPLS-DA) score plot model indicating separation between non-pregnant women (red) and pregnant women (green) both groups diagnosed with uterine factor problems, clustering with data on positive ion mode. Figure generated using MetaboAnalyst (www.metaboanalyst.com). The mass spectrometry analysis was performed in quintuplicate for each sample, therefore each patient is represented by 5 (five) dots.

**Figure 3:**
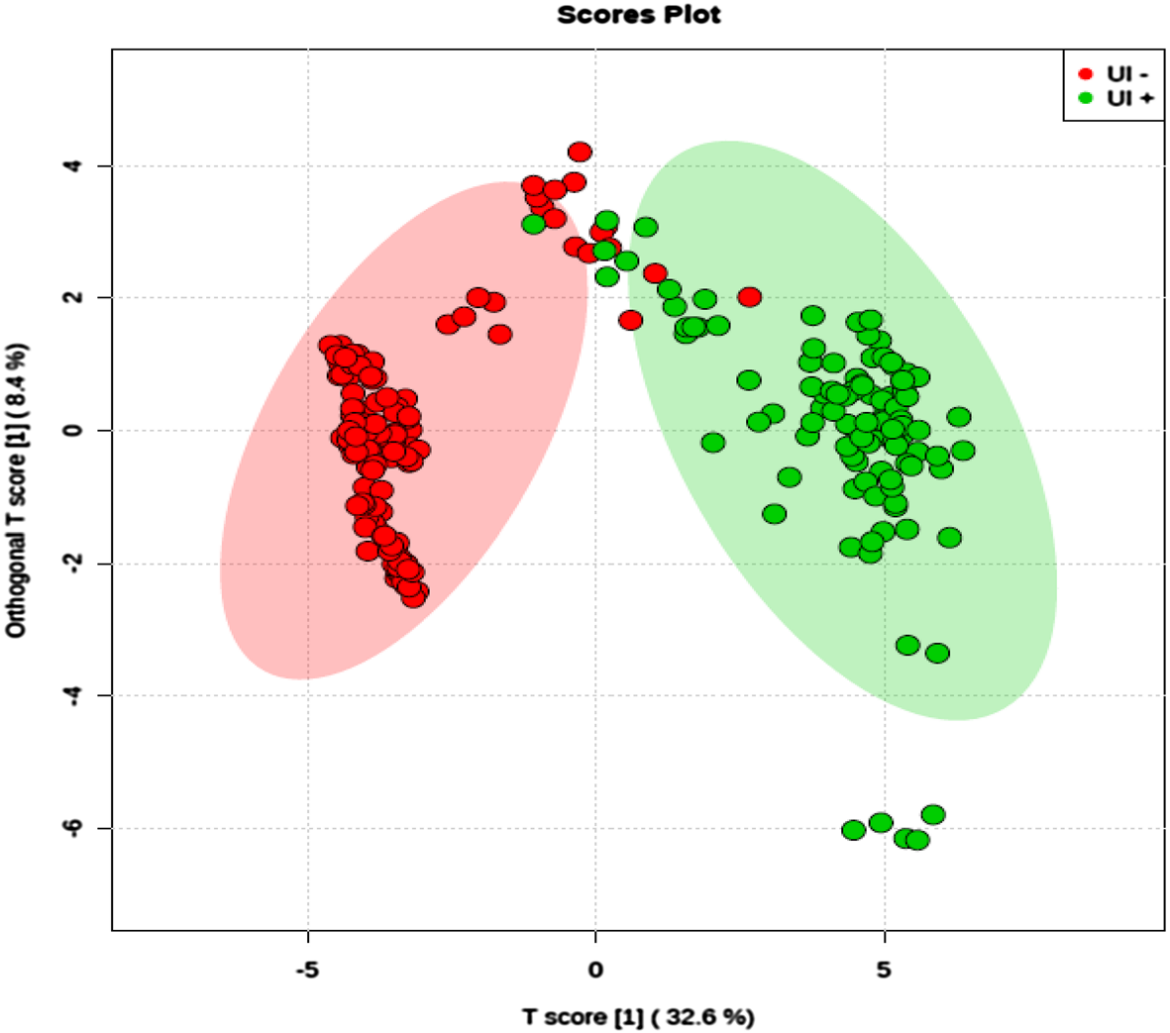
Unexplained Infertility Score Plot. Orthogonal partial least squares discriminant analysis (OPLS-DA) score plot model indicating separation between non-pregnant women (red) and pregnant women (green), both groups without a clinical diagnosis, clustering with data on positive ion mode. Figure generated using MetaboAnalyst (www.metaboanalyst.com). The mass spectrometry analysis was performed in quintuplicate for each sample, therefore each patient is represented by 5 (five) dots.

**Figure 4:**
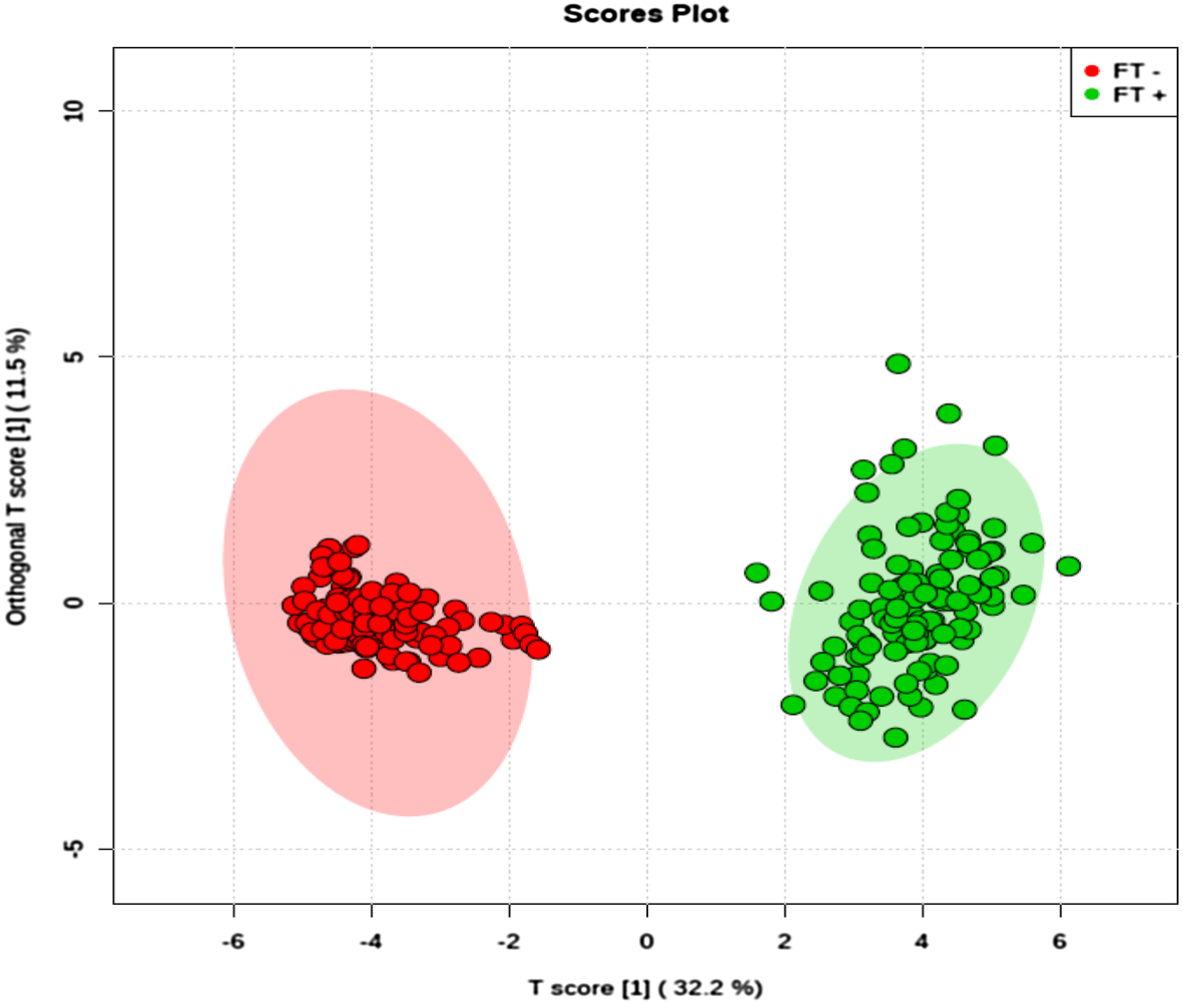
Tuboperitoneal Factor Score Plot. Orthogonal partial least squares discriminant analysis (OPLS-DA) score plot model indicating separation between non-pregnant women (red) and pregnant women (green), both groups with a tuboperitoneal factor diagnosis, clustering with data on positive ion mode. Figure generated using MetaboAnalyst (www.metaboanalyst.com). The mass spectrometry analysis was performed in quintuplicate for each sample, therefore each patient is represented by 5 (five) dots.

Results of statistical analysis of the data obtained from mass spectrometry indicated the significant differences in metabolite composition between IF follicular fluid and CT follicular fluid. The scores plot for endometriosis (Fig. 1), uterine factor (Fig. 2), unexplained infertility (Fig. 3), and tuboperitoneal factor (Fig. 4) groups demonstrate that the metabolic profiles of non-pregnant women are statistically different from those of women who were able to become pregnant undergoing the same treatment.

From a threshold value of 3.1 in VIP scores and AUC scores equal to or greater than 0.9, the biochemical markers for each infertile group were highlighted. The proposed prototype supported the election of two different biomarkers for the endometriosis group–PA 35:66, PA 37:7; six for the uterine factor group–GlcCerC(d18:0/20:0), Phosphatidylethanolamine, PC(16:0/16:0), PG(O-18:0/16:1(9Z)), PE(O-18:0/22:2(13Z,16Z)), and GluCer d18:1/24:0; one for the unexplained infertility–PI(O-16:0/15:1(9Z)); and one for the tuboperitoneal factor group–1-Hydroxyvitamin D3 3-d-glucopyranoside. The groups, experimental mass (*m/z)*, theoretic mass (*m/z)*, adducts, and names of all molecules are presented in Table 1.

**Table 1:** Chemical markers elected by Ortho-PLS-DA VIP score.

Group	Experimental mass (*m/z*)	Theoretical mass (*m/z*)	Molecule	Adducts	Metlin ID
Endometriosis	717.3910	717.3892	PA 35:6	M+K	81818
	727.4330	727.4309	PA 37:7	M+Na	81465
Uterine factor	758.6492	758.6504	GlcCerC(d18:0/20:0)	M+H	41616
	770.5699	770.5670	Phosphatidylethanolamine	M+Na	4193
	772.5225	772.5253	PC(16:0/16:0)	M+K	39306
	773.5112	773.5093	PG(O-18:0/16:1(9Z))	M+K	79883
	786.6401	786.6371	PE(O-18:0/22:2(13Z,16Z))	M+H	77538
	812.696	812.6974	GluCer d18:1/24:0	M+H	41612
Unexplained infertility	833.4607	833.4577	PI(O-16:0/15:1(9Z))	M+K	80184
Tuboperitoneal factor	599.3531	599.3554	1-Hydroxyvitamin D3 3-d-glucopyranoside	M+Na	42597

## Discussion

Clinical identification of the causal factor of infertility involves an extensive anamnesis of the couple’s reproductive health, hormonal, biochemical, and imaging tests that altogether can indicate possible causes that impair pregnancy. However, knowledge in this area is far from being exhausted, especially concerning the pathophysiological mechanism of clinical conditions associated with infertility.

Selecting good follicles for IVF procedures is of paramount importance for increasing the probability of a positive pregnancy outcome. Furthermore, the stimulation protocol must result in a minimum of three mature (or close to mature) follicles^18^. Although it is not consensual in the literature, it is believed that the largest follicles are more likely to contain mature oocytes that are capable of fertilization and, consequently, generate good embryos^19^. For these reasons, the three largest follicles were selected in this study.

The success rate of IVF is directly related to the number of embryos transferred and embryo quality. Although a lower number of transferred embryos decreases the probability of pregnancy, a higher number of transferred embryos increase the probability of multiple pregnancies^20^. According to the Assisted Reproductive Technology National Report, “the percentage of transfers that resulted in live births was highest (42%) when two embryos were transferred, however the percentage of multiple-infant births also was highest (34%)”^21^. To reduce the chance of multiple pregnancies, The American Society of Reproductive Medicine suggests that patients < 37 years old receive a single-embryo transfer, regardless of the embryo stage^22^. Therefore, the selection and quality of the embryo play a very important role in the fertilization process. Several different embryo selection criteria and embryo scoring systems have been described in the literature to evaluate the embryo with the most potential to achieve pregnancy. These systems may present some benefits such as accurate selection of embryos prior to transfer and reduction of the risk of multiple pregnancies^23^. Although each embryo scoring system differs concerning the selection of embryos and criteria for the assessment of embryo quality, in the majority of the cases, they explore the same characteristics such as inner cell mass, trophectoderm, and degree of expansion. Some simple grading systems only use one score to rank the overall appearance of the cleavage stage embryo. More complex systems use a formula to predict pregnancy outcomes regarding the appearance and development of an embryo^24^.

The most used and well-known grading system was implemented by the Society for Assisted Reproductive Technology (SART) and evaluates the quality of the embryos on days 3 (cleavage stage) or 5 (blastocyst stage) after egg retrieval and laboratory fertilization. This embryo score system is represented by two letters; the first indicates the development of the inner cell mass, and the second indicates the trophectoderm degree. The letters “A”, “B”, and “C” are used to represent the embryo quality potential, being “AA” the most potential and “CC” the least potential embryo. However, an “AA” embryo is not required to achieve pregnancy, as the majority of healthy babies are not graded “AA”^25^. Although embryo morphological scores are not directly linked to the developmental potential, they may be connected with the embryo implantation potential. Therefore, it must be carefully considered in IVF procedures. In this study, we performed a single-embryo transfer, and the best-graded embryo according to the SART system and patient's availability was chosen.

Clinical characteristics such as age, height, and weight need to be taken into consideration for IVF procedures. The age limit in this study was determined based on the decay of the ovarian’s reserve and quality in women older than 37 years old^26^. Although height and weight individually may not directly interfere with pregnancy outcomes, BMI may be related to pregnancy complications. Women with BMI values > 30 (obesity) are more likely to develop gestational diabetes, and hypertension, undergo cesarean sections, give birth to a baby who is large for gestational age, and generate low-quality embryos. In addition, BMI values < 18.5 are associated with an increased risk of preterm deliveries, low birth weight, anemia, and even non-ovulation^27^. Even though the BMI may not directly affect pregnancy outcomes, maintaining weight at normal levels in pregnant women could have significant beneficial effects on the health of both mother and child^28^.

### Endometriosis

Phosphatidic acids (PAs) are the simplest and most abundant phospholipids in cell membranes. PAs primary function is cell signaling; they act as a secondary messenger in several metabolic pathways^29^. In addition, PAs are the main precursors of other phospholipids, such as phosphatidylcholines (PC), phosphatidylethanolamines (PE), phosphatidylserine (PS), phosphatidylglycerol (PG), and phosphatidylinositol (PI). PAs are directly involved in several pathophysiological processes such as cell proliferation, inflammation, oncogenesis, phagocytosis, and apoptosis. The primary enzyme responsible for the production of PAs is phospholipase D (PLD), which acts by hydrolyzing the phosphodiester bonds of the glycerophospholipids^30^. Thus, in stress situations where PLD expression is exacerbated, there is an increase in the production of PAs, and it is strongly related to inflammatory processes. When PAs are overproduced, they affect tissues, causing diseases such as cancer, for example, in the brain, kidney, liver, breast, colon, and ovary^31^. Ovary cancer is directly related to negative pregnancy outcomes. Endometriosis, for instance, is an inflammatory disease prevalent in women of reproductive age and may cause abdominal pain and infertility-related complications. It is characterized by endometrial tissue implantation outside the uterine cavity, leading to a critical inflammatory process^32^, and may be associated with the accumulation of phospholipids.

Results presented in Fig. 1 demonstrate that the metabolic profiles of IF women are statistically different from CT women in the endometriosis subgroup. The statistical separation among groups confirms the existence of discriminative analytes associated with infertility problems. Two molecules, PA(35:6) and PA(37:7), were observed in more significant amounts in patients with endometriosis who did not become pregnant than in the control group (Table 1). The first molecule was described by Bai et al.^33^ as an intermediate of the PI molecule PI(20:5/15:1), which is a potential marker for cases of lymphoblastic leukemia. The second PA molecule identified is an intermediate for the marker described by Turkoglu et al.^34^ for ovarian cancer, TG(17:2/17:2/20:5).

In research conducted by Brinton et al.^35^, it was described that women who were previously diagnosed with endometriosis have a 90% higher risk of developing ovarian cancer and a 40% higher risk of developing hematopoietic cancer than women without this diagnosis. It is essential to understand that both endometriosis and uterine fibroids, another leading cause of infertility, have a replicative character, thereby resembling the behavior of cancer cells. PAs have been reported to exert apoptotic effects to promote cell proliferation and generate survival signals, which are directly linked to the proliferative capacity of endometriosis. Furthermore, PAs are related to cell mobility, resulting in endometrium migration and invasion^36^. Genetic alterations in endometrial cells are believed to be involved in the emergence of other types of cancer^37^. Thus, PAs can be described as markers arising from the infertility problems generated by the patient's condition, and endometriosis may be related to neoplastic processes owing to its proliferative capacity.

A metabolomics study conducted with peritoneal fluids of women with ovarian endometriosis and women without endometriosis observed that 10 metabolites had altered levels between the groups^38^, including PAs and PCs. Previously, the same research group had published the finding of elevated levels of PA, PC, and sphingomyelin in infertile patients suffering from endometriosis^39^. Thus, these previous studies corroborate our findings in the follicular fluid and reinforce PA as an infertility-related biomarker for the endometriosis subgroup. In this context, the markers found in this subgroup may be triggering biochemical pathways that are negatively affecting pregnancy outcomes in the IF group and may impair pregnancy outcomes by affecting directly the anatomy of the pelvis, adhesions, fallopian tubes, pelvic structures, immune system functioning, hormonal environment of the eggs, implantation, and egg quality^40^.

### Uterine factor

Uterine myomas (leiomyomata, fibroids) are a benign and the most common form of reproductive tract tumor, with a cumulative incidence of 50% in women of reproductive age^41^. Common symptoms from leiomyoma are heavy, painful menstrual bleeding, often leading to anemia, pelvic pressure and urinary frequency. However, there is evidence to conclude that the presence of myomas reduces the likelihood of achieving pregnancy^42^.

Results presented in Fig. 2 demonstrate that the metabolic profiles of IF women are statistically different from CT women in the uterine factor subgroup. The statistical separation among groups confirms the existence of discriminative analytes associated with infertility problems. Six molecules are proposed as markers of infertility in patients diagnosed within the uterine factor group (Table 1). PCs, PEs, and the enzymes involved in their metabolic pathways are characterized as the major structural components of eukaryotic cell membranes. These lipids act in several functions, play a leading role in cell signaling, growth regulation, and are currently being defined as potential markers for different types of cancers^43^. Excessive production of these three molecules (phosphatidylethanolamine, PC(16:0/16:0), and PE(O-18:0/22:2(13Z,16Z)) in women who could not achieve pregnancy suggests that the imbalance of essential and structural molecules affects the reproductive system.

The synthesis and biochemical pathways of these molecules are well established and represented by the Kennedy Pathway^44^, and the production of these lipids at abnormal levels is directly related to cases of lung, colon, breast, prostate, and ovarian cancer^45^. The inequality of these stimuli occurs owing to genetic and environmental factors and is directly related to increased levels of enzymes such as phosphatidylinositol 3-kinase (PI3K) and the abnormal activation of the Ras gene^46^. Several neoplasms analyzed by magnetic resonance spectroscopy indicated high concentrations of choline and ethanolamine phospholipid^47^. Furthermore, several enzymes involved in the Kennedy Pathway are related to carcinogenic processes and infertility-related complications^48^. Thus, the molecules found as markers of patients diagnosed with uterine abnormalities (phosphatidylethanolamine, PC(16:0/16:0) and PE(O-18:0/22:2(13Z,16Z)) agree with the data described in the literature, which are metabolites involved in processes that trigger the formation of cancer cells, which mightly affect pregnancy outcomes. Therefore, these three molecules found for this condition may confer a better understanding of the pathophysiological processes for this type of neoplasm and its relation to the success or failure of the pregnancy process.

Another molecule with a higher incidence in patients diagnosed with a uterine factor who could not become pregnant was PG(O-18:0/16:1(9Z)). PG is also a well-characterized class of phospholipids and is predominantly present in cellular membranes, being of vital importance in several functions and cellular structures. PGs have fundamental roles in cellular responses, including changes in membrane polarity, fluidity, flexibility, and morphology^49^. The synthesis and regulation of these phospholipids are the main factors responsible for homeostasis and regulation of the adaptive character of cells in stressful situations. Cardiolipin (CL), for example, is one of the most essential and abundant derivatives of PG, acting as a cellular response to environmental variations^50^. CL is predominantly found in the inner layer of the mitochondrial membrane and is a vital molecule owing to its function in the electron transport chain, acting on the efficiency of the cellular energy generation process.

CL also has a structural function, supporting several proteins involved in oxidative phosphorylation. By allowing ATP synthesis, the mitochondrial membrane is essential for the proper functioning of the organelle, and the non-regulation or alteration in levels of CL is associated with a series of conditions and diseases^51^. CL regulation in the mitochondrial membrane is also responsible for the transition from the resting state to a growth state. When this process is not regulated correctly, cells initially in a resting state begin to divide without adequate control, leading to the appearance of cancer cells. CL is also heavily involved in diseases such as Parkinson’s disease^52^. Women with Parkinson’s disease have been reported to have more infertility problems than healthy women owing to estrogen levels. The link for these two conditions might be CL^53^. Thus, phosphatidylglycerol homeostasis is crucial for cellular processes and vital functions, and may be closely related to infertility problems. Consequently, PG found in this study, in the uterine factor group, corroborates the data obtained in the literature, demonstrating that high levels of phosphatidylglycerol may be one of the possible factors responsible for the onset of infertility problems found in the studied patients.

This study demonstrated that some molecules ((GlcCer(d18:0/20:0)) and (GlcCer(d18:1/24:0)) are over expressed in the uterine factor group that could not get pregnant, demonstrating they are being accumulated and may be associated with infertility problems. Glucosylceramides (GlcCer) are derived from sphingolipids and considered essential structural components of cell membranes. This molecule class plays a significant role in biochemical routes that produce more complex GlcCer, such as lactosylceramide and gangliosides. GlcCers act as effector molecules in gene regulation, modulating cell growth, apoptosis, endocytosis, cell migration, senescence, and inflammatory processes^54^. The enzyme glucosylceramide synthase (GSL) regulates these molecules, acting as the balance between concentrations of ceramides and GlcCer. When the ratio between ceramides and GlcCer is not properly regulated owing to poor regulation of GSLs, cells undergo malignant and exacerbated cell growth. The accumulation of GlcCer at cellular levels can trigger tumor formation, especially in the uterus, ovaries, and breast^55^. However, this process is reversible, and previously cancerous cells can return to their normal development, making this class of molecules and its metabolic pathway an interesting therapeutic target for cancer evaluation studies^56^.

Although the biomarkers found in the follicular fluid of infertile patients with a uterine factor diagnosis are derived from different classes of molecules, they are all associated with abnormal cell growth and are present at a higher incidence in patients who could not become pregnant (IF group). Uterine fibroids, for instance, can distort the endometrial cavity making it harder for the implantation of the embryo; polyps can prevent implantation or even block the fallopian tubes; and adhesions are directly linked to the implantation process. The biomarkers found in the uterine factor group can act directly in these described metabolical pathways and are negatively related to pregnancy outcomes. These findings may allow a better understanding of the pathophysiological processes for this type of condition and its relationship with the success of pregnancy, improving the current therapeutic approaches for IVF procedures.

### Unexplained infertility

PI is a phospholipid and minor component in cell membranes, representing about 2–10% of the total phospholipid composition. However, this lipid class has a crucial role in regulating several cellular processes. PIs are directly involved in cell dynamics, traffic, and rearrangement. The inositol ring can be quickly phosphorylated in several positions, originating phosphoinositides (PIPns)^57^. Regulation of these PI subclasses is performed by PI-kinases and phosphatases, such as PI3Ks, an enzyme previously described as responsible for regulating PE and PC. PI3Ks belong to the family of lipid kinases responsible for regulating growth factors and cytokines that modulate and enable the signaling of cell proliferation, growth, survival, mobility, and metabolism processes, that is, the pathways most commonly activated in human cancer^58^.

According to one study^59^, alterations in the activation and regulation of these enzymes are responsible for causing abnormalities in the structure and function of the ovary, such as in the oocyte and follicle formation, and important for the activation and survival of primordial follicles, lowering the chances of a positive pregnancy outcome. Primordial follicles are the defined number of follicles found in mammalian ovaries and represent the entire reserve of the ovary during female reproductive life. Studies have demonstrated that the premature activation of PI3K in primordial oocyte signaling results in the premature activation of primordial follicles, leading to their exhaustion, and making pregnancy impossible. Changes in the activation and regulation of these enzymes have already been described in the literature as being responsible for causing abnormalities in the formation and function of the ovary. Considering that the number of oocytes is determined before the woman’s birth and that each woman’s reproductive life is dependent on this reserve, early recruitment and premature atresia of oocytes by deregulation of the PI3K pathway could lead to low-quality follicles and premature depletion of ovarian reserve.

Results presented in Fig. 3 demonstrate that the metabolic profiles of IF women are statistically different from CT women in the unexplained infertility subgroup. The statistical separation among groups confirms the existence of discriminative analytes associated with infertility problems. The statistical separation for women diagnosed with unexplained infertility, in particular, was harder to perform than that for the other three groups because all women in this group did not have the same assertive diagnosis for the same condition. As mentioned above, unexplained infertility is the lack of clinical diagnosis in women who have gone through all the standard investigations and tried to become pregnant and were not successful.

Fabi et al.^60^ recently demonstrated that PI3K has an important role in decidualization, an event in the uterine tissue, which affects the embryo’s implantation. Therefore, activation problems in this metabolic route are directly associated with infertility, as they negatively contribute to ovulation and nidation processes. Although the precise cause of infertility in this group remains uncertain, the presence of high levels of PI(16:0/15:1(9Z)) may indicate an accumulation and imbalance of PI3K levels in the IF group, negatively affecting pregnancy outcomes.

### Tuboperitoneal factor

Vitamin D3 has a multifactorial effect on the body, as it can regulate more than 1000 genes that directly affect various functions in the metabolism^61^. The most known and studied function of vitamin D3 is associated with the regulation of homeostasis between calcium and potassium concentrations at a cellular level. However, there is also a strong correlation in the literature with immune system regulation^62^, reproductive physiology, and bone mineralization. Changes in vitamin D3 levels are associated with several types of diseases, such as colon, breast, and skin cancer, and chronic inflammation^63^, although recent research also suggests vitamin D3 as a protective agent against many types of neoplasms^64^. The protective effect against cancer cells is associated with cell proliferation and differentiation, apoptosis, DNA repair, and inflammatory processes^65^. All diseases generated by changes in vitamin D3 levels have in common the interruption of endothelial stability and increased vascular leakage, often caused by injury or anatomical changes in the organ or tissue^66^. Vitamin D regulates a critical transcription factor to create the link between the embryo and the endometrium (HOXA-10), implying the possibility that vitamin D3 signals and contributes to the embryo's correct implementation and immune system tolerance^67^. Tuboperitoneal factor consists of morphological and anatomical changes in the fallopian tubes, causing chronic inflammatory processes and infertility problems. By regulating inflammatory processes, which may be related to infertility, and causing interruption of endothelial stability, the increase in the levels of 1-hydroxyvitamin D3 3-d-glucopyranoside corroborates the data found in the literature, which may lead to fertility problems.

Results presented in Fig. 4 demonstrate that the metabolic profiles of IF women are statistically different from CT women in the tuboperitoneal factor subgroup. The statistical separation among groups confirms the existence of discriminative analytes associated with infertility problems.

The infertility picture suggests that the balance of vitamin D is extremely important to women’s reproductive health^68^. Granulosa cells surrounding the oocyte contain receptors for vitamin D. Ovulatory dysfunctions are sometimes demonstrated in rats, as vitamin D regulates the production of anti-müllerian hormone (AMH), which is responsible for the follicular pool. Thus, some studies relate vitamin D with reasonable fertilization rates in ART cycles^69^ or presuppose that low vitamin D concentrations increase the chance of infertility.

Vitamin D supplementation is a common factor in the diet, and its widespread use has become increased in recent years. However, the most important data regarding vitamin D in follicular fluid were described by Anifandys et al.^70^, who reported that follicular fluid with high levels of vitamin D results in a reduction in embryo quality, which directly affects IVF outcomes. Therefore, vitamin D signals may contribute positively and negatively to pregnancy outcomes. The selection of 1-hydroxyvitamin D3 3-d-glucopyranoside as a possible marker for infertility in follicular fluid corroborates the data found in the literature; however, studies with more practical support are needed for the use of this biomarker.

## Conclusion

Our findings suggest that follicular fluid can be used to identify possible biomarkers that are negatively affecting pregnancy outcomes in female infertility conditions, such as uterine factor, endometriosis, tuboperitoneal factor, and unexplained infertility, thereby expanding the knowledge in this field. Based on these findings, we propose several altered biochemical pathways in the IF group, which are directly related to cell signaling, cell proliferation, inflammation, oncogenesis, and apoptosis, and may be reducing the efficacy of IVF procedures.

Follicular fluid is underused for diagnostic purposes and can be collected before the first round of IVF, given that it would be readily available during oocyte collection, resulting in no additional harm to the patient. The most notable data presented by this study are the potential of these biomarkers, which negatively affect pregnancy outcomes, for the more rapid diagnosis of some infertility-related diseases, leading to proper treatment, and increasing the chance of achieving pregnancy.

Our study provides new perspectives for the development of diagnostic assays using metabolomics. However, multicenter randomized studies should be conducted to validate our proposed method and establish the effectiveness of the markers. Nonetheless, our results pave the development of more rapid diagnostic methods, lowering the costs compared with standard techniques for infertility diagnosis and providing a better quality of life and timely results for patients.

## Data Availability

The datasets used and/or analyzed during the current study are available from the corresponding author on reasonable request.

## References

[1] Abdullah AA, Ahmed M, Oladokun A (2021). Prevalence of infertility in Sudan: A systematic review and meta-analysis. Qatar Med. J..

[2] de Faria DE, Grieco SC, de Barros SM (2012). The effects of infertility on the spouses’ relationship. Rev. Esc. Enferm. USP.

[3] Hanson B (2017). Female infertility, infertility-associated diagnoses, and comorbidities: A review. J. Assist. Reprod. Genet..

[4] Frith L, Blyth E (2014). Assisted reproductive technology in the USA: Is more regulation needed?. Reprod. Biomed. Online.

[5] Kettner LO, Henriksen TB, Bay B, Ramlau-Hansen CH, Kesmodel US (2015). Assisted reproductive technology and somatic morbidity in childhood: A systematic review. Fertil. Steril..

[6] Abrao MS, Muzii LMR, Marana R (2013). Anatomical causes of female infertility and their management. Int. J. Gynaecol. Obstet..

[7] Luttjeboer FY (2009). The value of medical history taking as risk indicator for tuboperitoneal pathology: A systematic review. BJOG.

[8] Marín HI, López IC, González AP, Huerta J (2022). Prevalencia de infecciones (Chlamydia, ureaplasma y Mycoplasma) en pacientes con factor tuboperitoneal alterado. Ginecol. Obstet. Mex..

[9] Cruz MSDDL, Buchanan EM (2022). Uterine fibroids: Diagnosis and treatment. Am. Fam. Phys..

[10] Al-Hendy A, Myers ER, Stewart E (2017). Uterine fibroids: Burden and unmet medical need. Semin. Reprod. Med..

[11] Ray A, Shah A, Gudi A, Homburg R (2012). Unexplained infertility: An update and review of practice. Reprod. Biomed. Online.

[12] Shen X (2017). Proteomic analysis of human follicular fluid associated with successful in vitro fertilization. Reprod. Biol. Endocrinol..

[13] Fortune JE (1994). Ovarian follicular growth and development in mammals. Biol. Reprod..

[14] Shah SH, Kraus WE, Newgard CB (2012). Metabolomic profiling for the identification of novel biomarkers and mechanisms related to common cardiovascular diseases: Form and function. Circulation.

[15] Barderas MG (2011). Metabolomic profiling for identification of novel potential biomarkers in cardiovascular diseases. J. Biomed. Biotechnol..

[16] Aronson JK, Ferner RE (2017). Biomarkers—a general review. Curr. Protoc. Pharmacol..

[17] Barbosa CP (2014). Low dose of rFSH [100 IU] in controlled ovarian hyperstimulation response: A pilot study. J. Ovarian Res..

[18] Evans MB (2020). Mature follicle count and multiple gestation risk based on patient age in intrauterine insemination cycles with ovarian stimulation. Obstet. Gynecol..

[19] Rosen MP (2008). A quantitative assessment of follicle size on oocyte developmental competence. Fertil. Steril..

[20] Nasiri N, Yazdi EP (2015). An overview of the available methods for morphological scoring of pre-implantation embryos in vitro fertilization. Cell J..

[21] Practice Committee of the American Society for Reproductive Medicine and the Practice Committee for the Society for Assisted Reproductive Technologies. Electronic address: ASRM@asrm.org. Guidance on the limits to the number of embryos to transfer: A committee opinion. *Fertil. Steril*. **116**, 3 (2021).

[22] Centers for Disease Control and Prevention, American Society for Reproductive Medicine, Society for Assisted Reproductive Technology. In *2014 Assisted Reproductive Technology National Summary Report. Atlanta, GA: US Dept of Health and Human Services*. 2016.

[23] Desai NN, Goldstein J, Rowland DY, Goldfarb JM (2000). Morphological evaluation of human embryos and derivation of an embryo quality scoring system specific for day 3 embryos: A preliminary study. Hum. Reprod..

[24] Cummins JM, Breen TM, Harrison KL, Shaw JM, Wilson LM, Hennessey JF (1986). A formula for scoring human embryo growth rates in in vitro fertilization: Its value in predicting pregnancy and in comparison with visual estimates of embryo quality. J. In Vitro Fert. Embryo Transf..

[25] Heitmann RJ (2013). The simplified SART embryo scoring system is highly correlated to implantation and live birth in single blastocyst transfers. J. Assist. Reprod. Genet..

[26] Chang Y (2018). Egg quality and pregnancy outcome in young infertile women with diminished ovarian reserve. Med. Sci. Monit..

[27] Verma A, Shrimali L (2012). Maternal body mass index and pregnancy outcome. J. Clin. Diagn. Res..

[28] Yang Z, Phung H, Freebairn L, Sexton R, Raulli A, Kelly P (2019). Contribution of maternal overweight and obesity to the occurrence of adverse pregnancy outcomes. Aust. N. Z. J. Obstet. Gynaecol..

[29] Zhukovsky MA, Filograna A, Luini A, Corda D, Valente C (2019). Phosphatidic acid in membrane rearrangements. FEBS Lett..

[30] Kang DW, Choi KY, Min DS (2014). Functional regulation of phospholipase D expression in cancer and inflammation. J. Biol. Chem..

[31] Liu Y, Su Y, Wang X (2013). Phosphatidic acid-mediated signaling. Adv. Exp. Med. Biol..

[32] Burney RO, Giudice LC (2012). Pathogenesis and pathophysiology of endometriosis. Fertil. Steril..

[33] Bai Y, Zhang H, Sun X, Sun C, Ren L (2014). Biomarker identification and pathway analysis by serum metabolomics of childhood acute lymphoblastic leukemia. Clin. Chim. Acta.

[34] Turkoglu O (2016). Metabolomics of biomarker discovery in ovarian cancer: A systematic review of the current literature. Metabolomics.

[35] Brinton LA, Gridley G, Persson I, Baron J, Bergqvist A (1997). Cancer risk after a hospital discharge diagnosis of endometriosis. Am. J. Obstet. Gynecol..

[36] Li J (2018). Discovery of phosphatidic acid, phosphatidylcholine, and phosphatidylserine as biomarkers for early diagnosis of endometriosis. Front. Physiol..

[37] Meola J (2010). Differentially expressed genes in eutopic and ectopic endometrium of women with endometriosis. Fertil. Steril..

[38] Vouk K, Ribič-Pucelj MR, Adamski J, Rižner TL (2016). Altered levels of acylcarnitines, phosphatidylcholines, and sphingomyelins in peritoneal fluid from ovarian endometriosis patients. J. Steroid Biochem. Mol. Biol..

[39] Vouk K (2012). Discovery of phosphatidylcholines and sphingomyelins as biomarkers for ovarian endometriosis. Hum. Reprod..

[40] Gallová UZ, Bouse V, Svábek L, Turek J, Rokyta Z (2002). Endometriosis in reproductive immunology. Am. J. Reprod. Immunol..

[41] Baird DD, Dunson DB, Hill MC, Cousins D, Schectman JM (2003). High cumulative incidence of uterine leiomyoma in black and white women: Ultrasound evidence. Am. J. Obstet. Gynecol..

[42] Practice Committee of the American Society for Reproductive Medicine. Removal of myomas in asymptomatic patients to improve fertility and/or reduce miscarriage rate: A guideline. *Fertil. Steril*. **108**, 416–425 (2017). 10.1016/j.fertnstert.2017.06.034 28865538

[43] Cataldi T (2013). Lipid profiling of follicular fluid from women undergoing IVF: Young poor ovarian responders versus normal responders. Hum. Fertil. (Camb.).

[44] Gibellini F, Smith TK (2010). The Kennedy pathway—de novo synthesis of phosphatidylethanolamine and phosphatidylcholine. IUBMB Life.

[45] Li K, Gray BD, Pak KY, Ng CK (2019). Targeting phosphatidylethanolamine and phosphatidylserine for imaging apoptosis in cancer. Nucl. Med. Biol..

[46] Zaravinos A (2017). Oncogenic RAS: From its activation to its direct targeting. Crit. Rev. Oncog..

[47] Sonkar K (2019). Focus on the glycerophosphocholine pathway in choline phospholipid metabolism of cancer. NMR Biomed..

[48] Cheng M, Bhujwalla ZM, Glunde K (2016). Targeting phospholipid metabolism in cancer. Front. Oncol..

[49] Frentzen M (2004). Phosphatidylglycerol and sulfoquinovosyldiacylglycerol: Anionic membrane lipids and phosphate regulation. Curr. Opin. Plant Biol..

[50] Nakagawa Y (2013). Metabolism and biological function of cardiolipin. Yakugaku Zasshi.

[51] Ahmadpour ST, Mahéo K, Servais S, Brisson L, Dumas JF (2020). Cardiolipin, the mitochondrial signature lipid: Implication in cancer. Int. J. Mol. Sci..

[52] Gilmozzi V (2020). Interaction of alpha-synuclein With lipids: Mitochondrial cardiolipin as a critical player in the pathogenesis of Parkinson’s disease. Front. Neurosci..

[53] Klatt-Schreiner K (2020). High glucosyl ceramides and Low anandamide Contribute to Sensory Loss and Pain in Parkinson’s disease. Mov. Disord..

[54] Messner MC, Cabot MC (2010). Glucosylceramide in humans. Adv. Exp. Med. Biol..

[55] Dunn WB (2012). The metabolome of human placental tissue: Investigation of first trimester tissue and changes related to preeclampsia in late pregnancy. Metabolomics.

[56] Schömel N (2019). UGCG influences glutamine metabolism of breast cancer cells. Sci. Rep..

[57] Poccia D, Larijani B (2009). Phosphatidylinositol metabolism and membrane fusion. Biochem. J..

[58] Ellis H, Ma CX (2019). PI3K inhibitors in breast cancer therapy. Curr. Oncol. Rep..

[59] Makker A, Goel MM, Mahdi AA (2014). PI3K/PTEN/Akt and TSC/mTOR signaling pathways, ovarian dysfunction, and infertility: An update. J. Mol. Endocrinol..

[60] Fabi F (2017). Regulation of the PI3K/Akt pathway during decidualization of endometrial stromal cells. PLoS ONE.

[61] Vilarino FL (2022). Análise do polimorfismo Fok1 do gene VDR em mulheres inférteis com Endometriose. Rev. Bras. Ginecol. Obstet..

[62] Cantorna MT, Mahon BDC (2004). Mounting evidence for vitamin D as an environmental factor affecting autoimmune disease prevalence. Exp. Biol. Med. (Maywood).

[63] Bjelakovic G (2014). Vitamin D supplementation for prevention of mortality in adults. Cochrane Database Syst. Rev..

[64] Ng K (2019). Effect of high-dose vs standard-dose vitamin D3 supplementation on progression-free survival Among patients With advanced or metastatic colorectal cancer: The SUNSHINE randomized clinical trial. JAMA.

[65] O’Shea SJ, Davies JR, Newton-Bishop JA (2016). Vitamin D, vitamin A, the primary melanoma transcriptome and survival. Br. J. Dermatol..

[66] Gibson CC (2015). Dietary vitamin D and its metabolites non-genomically stabilize the endothelium. PLoS ONE.

[67] Curtis-Hewitt SC, Goulding EH, Eddy EM, Korach KS (2002). Studies using the estrogen receptor alpha knockout uterus demonstrate that implantation but not decidualization-associated signaling is estrogen dependent. Biol. Reprod..

[68] Dabrowski FA, Grzechocinska B, Wielgos M (2015). The role of vitamin D in reproductive health—a Trojan horse or the Golden Fleece?. Nutrients.

[69] Abadia L (2016). Serum 25-hydroxyvitamin D concentrations and treatment outcomes of women undergoing assisted reproduction. Am. J. Clin. Nutr..

[70] Anifandis GM (2010). Prognostic value of follicular fluid 25-OH vitamin D and glucose levels in the IVF outcome. Reprod. Biol. Endocrinol..

